# Modulation of Advanced Glycation End Products and Oxidative Stress by Hesperetin-7-*O*-Glucoside and Diosmetin-7-*O*-Glucoside Complexed with Cyclodextrins

**DOI:** 10.3390/ph19081224

**Published:** 2026-08-04

**Authors:** José Moreira Tavares Neto, Bianca Soriano dos Anjos, Joyce Lopes Macedo, José Otávio Carvalho Sena de Almeida, Clailson da Silva Pinheiro, Fernando Aécio de Amorim Carvalho, Maria do Carmo de Carvalho e Martins, Leonardo da Rocha Sousa, Junya Kobayashi, Damião Pergentino de Sousa, Daniel Dias Rufino Arcanjo

**Affiliations:** 1 LAFMOL–Laboratory of Functional and Molecular Studies in Physiopharmacology, Department of Biophysics and Physiology, Federal University of Piaui, Teresina 64049-550, Brazil; jose.mtavares@ufpi.edu.br (J.M.T.N.); otavios.almeida@hotmail.com (J.O.C.S.d.A.); leonardorocha@ifpi.edu.br (L.d.R.S.); 2 BioLeish–Advanced Research in Leishmania and Alternative Models, Medicinal Plants Research Center, Federal University of Piaui, Teresina 64049-550, Brazil; bianca.sanjos@ufpi.edu.br (B.S.d.A.); famorim@ufpi.edu.br (F.A.d.A.C.); 3 Laboratory of Physiological Sciences, Department of Biophysics and Physiology, Federal University of Piaui, Teresina 64049-550, Brazil; joycelopes385@gmail.com (J.L.M.); carminhamartins@ufpi.edu.br (M.d.C.d.C.e.M.); 4 Department of Information, Environment, Health and Food Production, Federal Institute of Piauí, Teresina 64000-040, Brazil; 5 Taiyo Kagaku Co., Ltd., Yokkaichi, 1-3 Takaramachi, Yokkaichi 510-0844, Mie, Japan; jukobayashi@taiyokagaku.co.jp; 6 Department of Pharmaceutical Sciences, Federal University of Paraíba, João Pessoa 58051-900, Brazil; dps2@academico.ufpb.br

**Keywords:** hyperglycemia, oxidative stress, advanced glycation end products, flavonoids, cyclodextrins

## Abstract

**Background/Objectives:** Chronic complications of diabetes mellitus are closely associated with increased oxidative stress and the formation of advanced glycation end products (AGEs). This study aimed to investigate the antioxidant and antiglycation potential of hesperetin-7-*O*-glucoside (HCD) and diosmetin-7-*O*-glucoside (DCD) formulations complexed with cyclodextrins, using in vitro and in silico experimental models to evaluate their efficacy in mitigating hyperglycemia-induced molecular damage. **Methods**: Antioxidant activity was assessed using chemical and erythrocyte-based oxidative stress models, whereas antiglycation activity was evaluated in BSA–fructose, BSA–methylglyoxal, and arginine–methylglyoxal models. **Results**: Both formulations showed measurable antioxidant effects, with concentration-dependent behavior observed in some of the evaluated assays. In the DPPH assay, HCD and DCD achieved maximum inhibition values of 30.71% and 26.61%, respectively. Furthermore, DCD exhibited higher total antioxidant capacity (102.80 µg vitamin C equivalents/mL) and nitric oxide scavenging activity (37.89%) than HCD. In a cellular model, both formulations (200 µg/mL) significantly reduced AAPH-induced hemolysis, with DCD providing superior protection (7.05% vs. 16.63% for HCD). Under oxidative stress induced by high glucose concentration in erythrocytes, HCD and DCD reduced non-protein thiol levels, and HCD significantly increased catalase enzyme activity. Regarding antiglycation activity, DCD demonstrated superior efficacy relative to HCD in the BSA-fructose system, achieving 43.23% inhibition. DCD also displayed concentration-dependent inhibition of fructosamine formation (up to 47.99%) and BSA glycation by methylglyoxal (up to 45.20%). Both formulations significantly reduced carbonylated protein levels and preserved free thiol groups. In the arginine–methylglyoxal model, DCD and HCD reached 44.03% and 48.82% inhibition, respectively. Molecular docking revealed high binding affinity of both flavonoids to the protein active site (−9.00 kcal/mol for hesperetin-7-*O*-glucoside and −9.04 kcal/mol for diosmetin-7-*O*-glucoside), suggesting a structural protective role. **Conclusions**: The HCD and DCD formulations demonstrated antioxidant and antiglycation activities that may contribute to attenuating molecular alterations associated with chronic hyperglycemia through complementary mechanisms, including antioxidant effects, protection against protein carbonylation, and inhibition of glycation. While these findings highlight the potential of the evaluated formulations, additional mechanistic and in vivo studies are required to establish their pharmacological applicability.

## 1. Background

Diabetes mellitus (DM) represents one of the major metabolic disorders of the present, characterized by persistent hyperglycemia and a high incidence of microvascular and macrovascular complications [1,2]. At the molecular level, the progression of these complications is strongly associated with excessive formation of advanced glycation end products (AGEs) and increased oxidative stress, interdependent and bidirectionally amplified processes that play a central role in the pathophysiology of the disease.

Non-enzymatic glycation occurs through the Maillard reaction between reducing sugars or their reactive metabolites and free amino groups of proteins, initially leading to the formation of Amadori products and subsequently to stable and structurally complex AGEs [3,4]. Under chronic hyperglycemic conditions, the rate of this reaction is significantly enhanced, promoting conformational alterations, functional loss, and the formation of protein cross-links.

Concurrently, high intracellular glucose levels favor the production of reactive oxygen species (ROS) through multiple metabolic pathways, including mitochondrial dysfunction and glucose auto-oxidation [5,6]. The resulting oxidative stress not only causes direct damage to lipids, proteins, and DNA but also accelerates glycation steps, promoting the conversion of intermediates into AGEs via glyco-oxidation mechanisms. Among these reactive glycation intermediates, methylglyoxal (MGO) stands out as a key AGE precursor and mediator of carbonyl stress. Its high reactivity with arginine, lysine, and cysteine residues induces rapid protein modifications, intensifying both AGE formation and redox imbalance [7,8]. Furthermore, AGE formation amplifies oxidative stress through activation of the receptor for advanced glycation end products (RAGE), triggering pro-inflammatory and pro-oxidant cascades that perpetuate cellular damage [9,10]. This bidirectional cycle establishes a chronic state of redox dysfunction, particularly relevant in tissues highly exposed to circulating glucose, such as vascular endothelium and erythrocytes.

In this integrated scenario of glycation and oxidative stress, effective therapeutic strategies should ideally act on multiple targets, simultaneously reducing AGE formation, carbonyl stress, and oxidative damage. Synthetic compounds such as aminoguanidine have demonstrated experimental potential for inhibiting AGE formation; however, safety-related limitations have restricted their clinical application [11]. Natural flavonoids have emerged as promising candidates in this context due to their capacity to neutralize free radicals, chelate metal ions, scavenge reactive carbonyl species, and protect protein structures against oxidative and glycative modifications [12,13,14]. Among these compounds, hesperetin-7-*O*-glucoside and diosmetin-7-*O*-glucoside are notable, with structural differences that may influence their antioxidant and antiglycation performance. Nevertheless, limitations related to solubility and bioavailability may compromise the pharmacological efficacy of these flavonoids.

Cyclodextrin-based inclusion complexes have therefore been proposed as a formulation strategy to improve the physicochemical stability, solubility, and biopharmaceutical performance of flavonoids [15,16]. However, despite these potential advantages, the antioxidant and antiglycation properties of hesperetin-7-*O*-glucoside and diosmetin-7-*O*-glucoside formulated as cyclodextrin inclusion complexes remain poorly characterized. In particular, studies combining complementary antioxidant assays with multiple glycation and glyco-oxidation models to provide a comprehensive evaluation of these formulations are still scarce.

Accordingly, the present study aimed to investigate the antioxidant and antiglycation potential of hesperetin-7-*O*-glucoside and diosmetin-7-*O*-glucoside formulations complexed with cyclodextrins, evaluating their capacity to modulate oxidative stress and AGE formation in in vitro experimental models, including methylglyoxal-mediated systems and erythrocyte protection assays, as well as exploring molecular interactions with proteins through docking studies.

## 2. Results

### 2.1. Antioxidant Activity in Chemical Models

#### 2.1.1. Effect of the Formulations on DPPH Radical Scavenging

**Figure 1:**
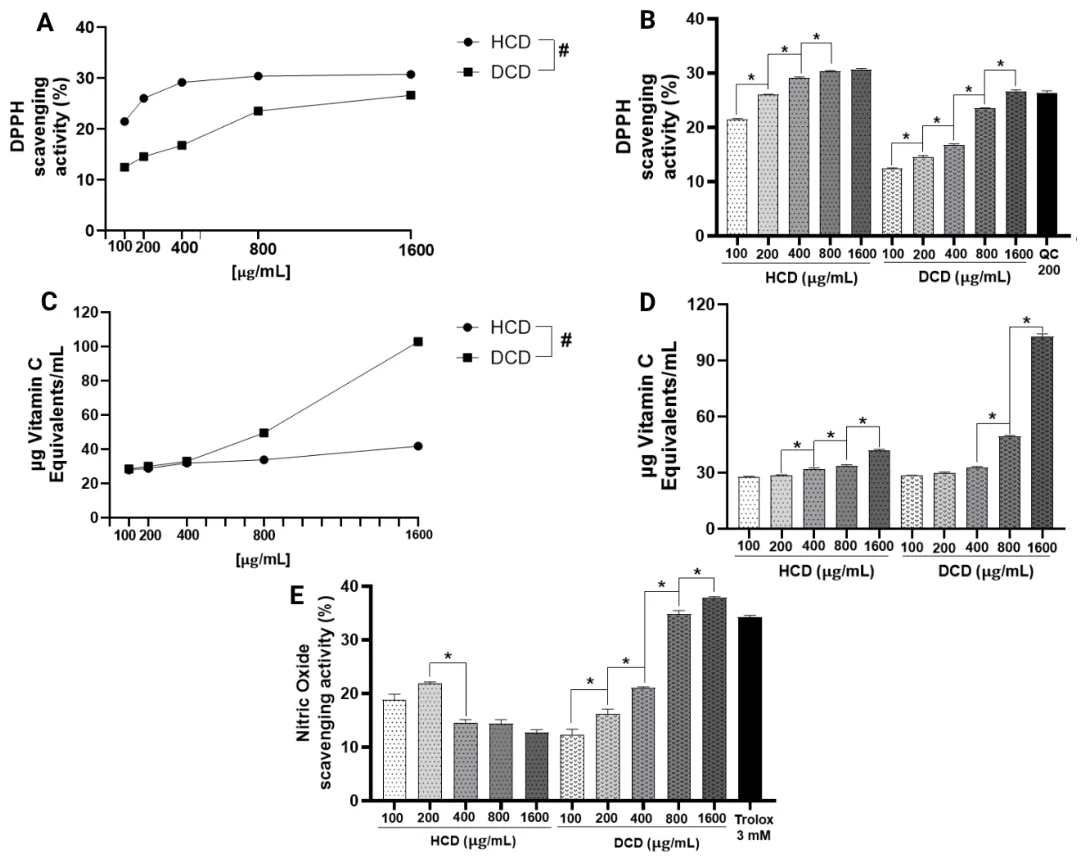
(**A**) Concentration–response curve of the formulations in DPPH radical scavenging. (**B**) Percentage of DPPH radical scavenging activity of the formulations at different concentrations and of quercetin. (**C**) Concentration–response curve of the formulations in total antioxidant capacity (TAC). (**D**) Micrograms of vitamin C equivalents per mL of the formulations at different concentrations. (**E**) Percentage of nitric oxide (NO) radical scavenging activity of the formulations at different concentrations and of Trolox. Data are expressed as mean ± SEM of three technical replicates. # *p* < 0.05 compared to HCD; * *p* < 0.05 compared to the previous concentration of the same formulation.

The HCD and DCD formulations exhibited concentration-dependent DPPH radical scavenging activity over the range of 100 to 1600 μg/mL (Figure 1A,B). The HCD formulation demonstrated significantly higher antioxidant activity compared to DCD (*p* < 0.05), reaching a maximum inhibition of 30.71 ± 0.149% at 1600 μg/mL, whereas DCD showed 26.61 ± 0.335% at the same concentration. Quercetin (200 μg/mL), used as a positive control, displayed a scavenging activity of 26.37 ± 0.369%.

#### 2.1.2. Total Antioxidant Capacity (TAC) of the Formulations

The total antioxidant capacity of the formulations was evaluated using the phosphomolybdate method. Both formulations (HCD and DCD) exhibited increased antioxidant activity, as a function of concentration, showing a concentration-dependent response (Figure 1C,D). For the HCD formulation, a significant increase was observed starting at 200 μg/mL, whereas for DCD, statistical significance became evident from 400 μg/mL (*p* < 0.05).

Compared with HCD, the DCD formulation displayed a significantly higher total antioxidant capacity (*p* < 0.05), reaching a maximum value of 102.8 ± 1.409 µg vitamin C equivalents/mL at 1600 μg/mL. At the same concentration, HCD showed 41.86 ± 0.585 µg vitamin C equivalents/mL (Figure 1C).

#### 2.1.3. Effect of the Formulations on Nitric Oxide (NO) Radical Scavenging

The DCD formulation exhibited high NO radical scavenging activity, with a significantly concentration-dependent response, reaching a maximum effect of 37.89 ± 0.202% at the highest tested concentration. In contrast, the HCD formulation showed lower scavenging capacity, without a clearly concentration-dependent profile, reaching a maximum value of 21.87 ± 0.309%, which was significantly lower than that observed for DCD and the positive control Trolox (750 μg/mL), which exhibited an activity of 34.27 ± 0.309% (Figure 1E).

### 2.2. Antioxidant Activity in a Cellular Model of Oxidative Stress

#### 2.2.1. Anti-Hemolytic Activity of the Formulations Against AAPH

**Figure 2:**
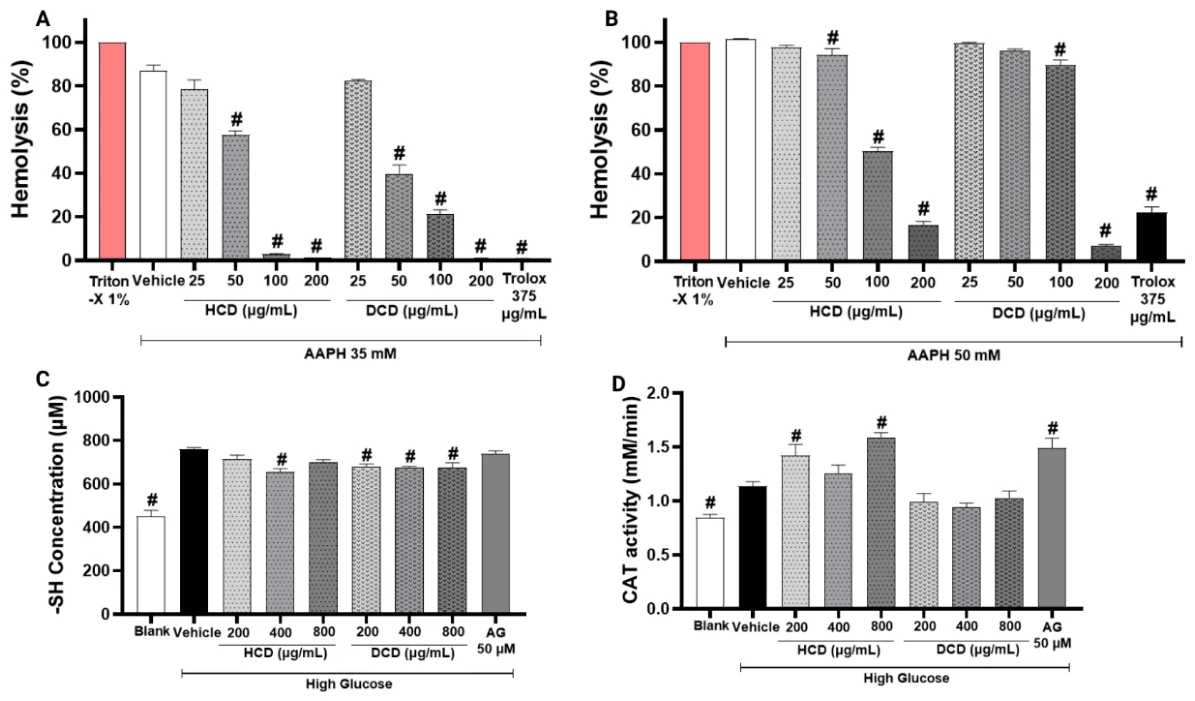
(**A**) Anti-hemolytic activity of the formulations and Trolox against AAPH-induced hemolysis at 35 mM. (**B**) Anti-hemolytic activity of the formulations and Trolox against AAPH-induced hemolysis at 50 mM. (**C**) Non-protein thiol group levels under erythrocyte oxidative stress induced by high glucose concentration and treated with the formulations or aminoguanidine. (**D**) Catalase enzyme activity under erythrocyte oxidative stress induced by high glucose concentration and treated with the formulations or aminoguanidine. Data are expressed as mean ± SEM of independent biological replicates (*n* = 3–5). # *p* < 0.05 compared to the vehicle group.

The anti-hemolytic activity of the HCD and DCD formulations was evaluated in erythrocytes subjected to oxidative stress induced by AAPH (35 and 50 mM). Incubation with AAPH plus vehicle resulted in pronounced hemolysis, reaching 87.01 ± 2.532% (35 mM) and 99.70 ± 0.133% (50 mM), confirming the effectiveness of the experimental model (Figure 2A,B).

Under the 35 mM AAPH condition (Figure 2A), both formulations significantly reduced hemolysis in a concentration-dependent manner (*p* < 0.05). The HCD formulation decreased hemolysis percentages to 3.06 ± 0.156% and 1.31 ± 0.025% at 100 and 200 µg/mL, respectively, approaching basal levels. For the DCD formulation, only the 200 µg/mL concentration exhibited a similar effect, reducing hemolysis to 1.06 ± 0.110%.

Under the higher oxidative stress condition (50 mM AAPH; Figure 2B), both formulations maintained significant and concentration-dependent anti-hemolytic activity (*p* < 0.05). At 200 µg/mL, hemolysis percentages were reduced to 16.63 ± 1.651% (HCD) and 7.05 ± 0.811% (DCD). These values were lower than those observed for the positive control Trolox (375 µg/mL), which exhibited 22.48 ± 2.425% hemolysis.

#### 2.2.2. Effects of the Formulations on Oxidative Stress Markers Induced by High Glucose Concentration

The antioxidant activity of the formulations was further evaluated in an erythrocyte oxidative stress model induced by high glucose concentration, through the determination of non-protein thiol (NPSH) levels (Figure 2C) and catalase (CAT) enzyme activity (Figure 2D).

The high glucose + vehicle group exhibited a significant increase in NPSH levels (761.10 ± 5.693 µM) compared to the blank group (449.20 ± 28.790 µM) (*p* < 0.05). Regarding the treated groups, all concentrations of the DCD formulation (200, 400, and 800 µg/mL) and HCD (400 µg/mL) significantly reduced NPSH levels compared to the high-glucose + vehicle group (*p* < 0.05).

Concerning catalase activity, the high-glucose + vehicle group (1.13 ± 0.042) showed a significant increase compared to the blank group (0.84 ± 0.029). In the treated groups, the HCD formulation at concentrations of 200 µg/mL (1.42 ± 0.102) and 800 µg/mL (1.59 ± 0.044), as well as the aminoguanidine-treated group (50 µM) (1.49 ± 0.090), significantly increased catalase activity compared to the high glucose + vehicle group (Figure 2D).

### 2.3. Antiglycation Activity of the Formulations

#### 2.3.1. Antiglycation Activity in the BSA–Fructose Model

**Figure 3:**
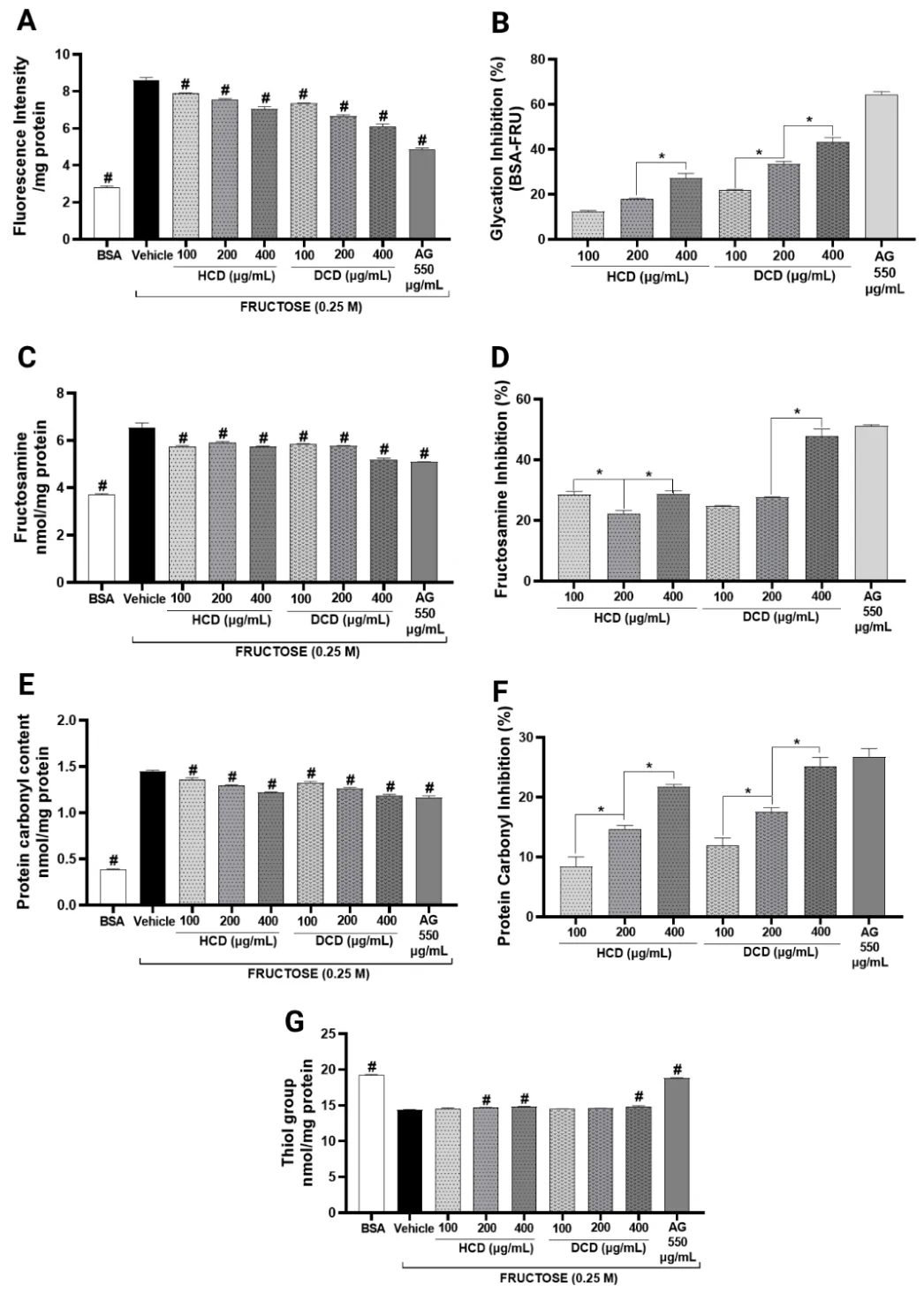
(**A**) Measurement of fluorescence intensity. (**B**) Percentage inhibition of fluorescent AGE formation. (**C**) Fructosamine levels in nmol/mg of protein. (**D**) Percentage inhibition of fructosamine formation. (**E**) Carbonylated protein concentration in nmol/mg of protein. (**F**) Percentage inhibition of protein carbonyl group formation. (**G**) Free thiol group concentration in nmol/mg of protein. Data are expressed as mean ± SEM of three technical replicates. # *p* < 0.05 compared to the vehicle group; * *p* < 0.05 compared to the previous concentration of the same formulation.

The fructose + vehicle group exhibited a significant increase in fluorescence (8.62 ± 0.124 AFU/mg) compared to non-glycated BSA (2.81 ± 0.065 AFU/mg), confirming the induction of glycation (Figure 3A).

HCD and DCD significantly reduced fluorescence at all evaluated concentrations (100–400 µg/mL) (*p* < 0.05). DCD exhibited a concentration-dependent effect, achieving a maximum inhibition of 43.23 ± 1.989%, whereas HCD reached 27.21 ± 2.115%. Aminoguanidine (550 µg/mL) showed the highest inhibition (64.52 ± 1.168%) (Figure 3B).

#### Effects of the Formulations on Fructosamine Levels

The fructose + vehicle group exhibited a significant increase in fructosamine levels (6.56 ± 0.180 nmol/mg) compared to non-glycated BSA (3.70 ± 0.047 nmol/mg) (Figure 3C).

The HCD and DCD formulations, as well as aminoguanidine, significantly reduced fructosamine formation (*p* < 0.05). DCD showed a concentration-dependent effect, with inhibition ranging from 27.63 ± 0.201% (200 µg/mL) to 47.99 ± 2.165% (400 µg/mL). HCD exhibited similar inhibition percentages across the tested concentrations, without a clearly concentration-dependent pattern. Aminoguanidine (550 µg/mL) achieved 51.25 ± 0.363% inhibition (Figure 3D).

#### Effects of the Formulations on Carbonylated Protein and Free Thiol Group Levels

The fructose + vehicle group showed a significant increase in carbonylated protein levels (1.45 ± 0.007 nmol/mg) compared to non-glycated BSA (0.38 ± 0.003 nmol/mg) (Figure 3E). Both HCD and DCD significantly reduced this parameter in a concentration-dependent manner (*p* < 0.05). Maximum inhibition was 21.75 ± 0.376% for HCD and 25.24 ± 1.422% for DCD, values similar to those observed for aminoguanidine (26.79 ± 1.351%) (Figure 3F).

The fructose + vehicle group exhibited a significant decrease in free thiol levels (14.40 ± 0.028 nmol/mg) compared to non-glycated BSA (19.25 ± 0.084 nmol/mg), indicating protein oxidation induced by glycation. The HCD formulation (200 and 400 µg/mL) and DCD (400 µg/mL) significantly mitigated the loss of free thiols (*p* < 0.05). Aminoguanidine (550 µg/mL) maintained levels close to those of the non-glycated BSA group (Figure 3G).

#### 2.3.2. Antiglycation Activity of the Formulations in the BSA–Methylglyoxal Model

**Figure 4:**
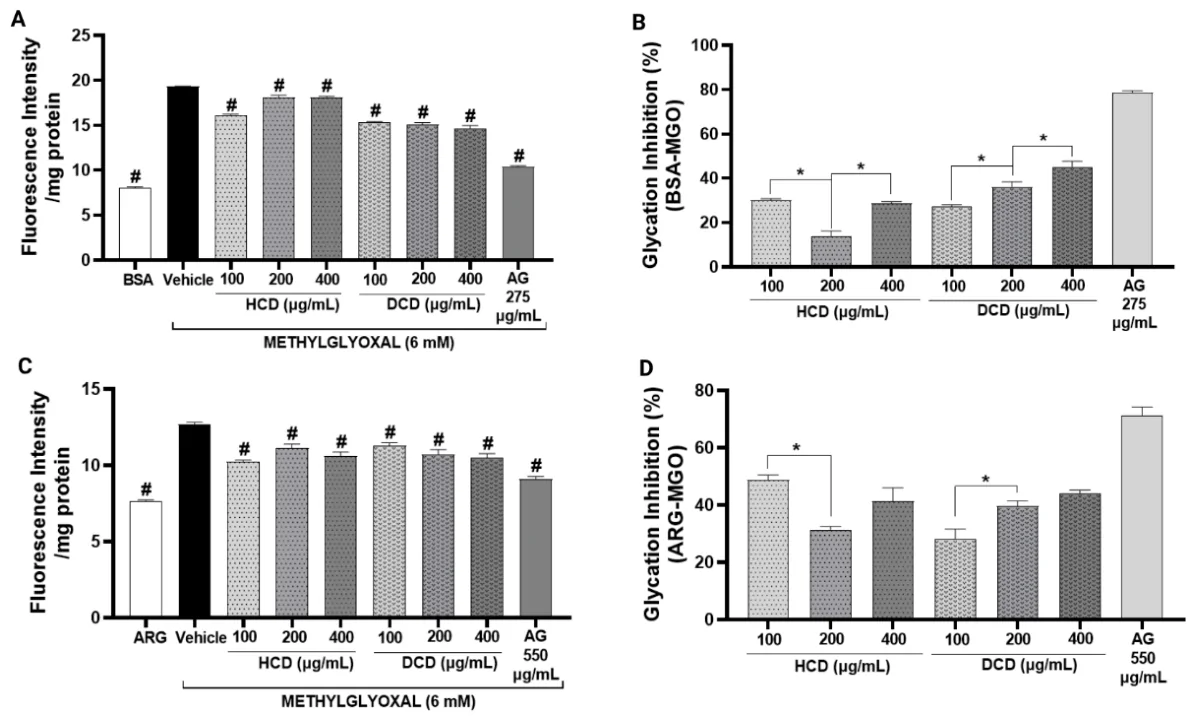
(**A**) Measurement of fluorescence intensity in the BSA–MGO model. (**B**) Percentage inhibition of fluorescent AGE formation in the BSA–MGO model. (**C**) Measurement of fluorescence intensity in the ARG–MGO model. (**D**) Percentage inhibition of fluorescent AGE formation in the ARG–MGO model. Data are expressed as mean ± SEM of three technical replicates. # *p* < 0.05 compared to the vehicle group; * *p* < 0.05 compared to the previous concentration of the same formulation.

The methylglyoxal + vehicle group exhibited a significant increase in fluorescence (19.32 ± 0.032 AFU/mg) compared to non-glycated BSA (8.02 ± 0.130 AFU/mg), confirming the induction of glycation (Figure 4A).

HCD and DCD significantly reduced fluorescence at all evaluated concentrations (100–400 µg/mL) (*p* < 0.05). DCD exhibited a concentration-dependent effect, with inhibition ranging from 27.28 ± 0.807% to 45.20 ± 2.522%. HCD showed similar inhibition percentages across the tested concentrations, without a clearly concentration-dependent pattern. Aminoguanidine (275 µg/mL) produced the highest inhibition (78.79 ± 0.717%) (Figure 4B).

#### 2.3.3. Antiglycation Activity of the Formulations in the Arginine–Methylglyoxal Model

The methylglyoxal + vehicle group exhibited a significant increase in fluorescence (12.72 ± 0.104 AFU/mg) compared to non-glycated arginine (7.66 ± 0.063 AFU/mg), confirming the induction of glycation (Figure 4C).

HCD and DCD significantly reduced AGE formation (*p* < 0.05). DCD showed a concentration-dependent effect, with inhibition ranging from 28.20 ± 3.454% to 44.03 ± 1.281%. HCD exhibited the highest inhibition at 100 µg/mL (48.82 ± 1.717%), without a concentration-dependent pattern. Aminoguanidine (550 µg/mL) produced the highest inhibition (71.25 ± 2.985%) (Figure 4D).

#### 2.3.4. Molecular Interaction of Hesperetin-7-*O*-Glucoside and Diosmetin-7-*O*-Glucoside with BSA

**Figure 5:**
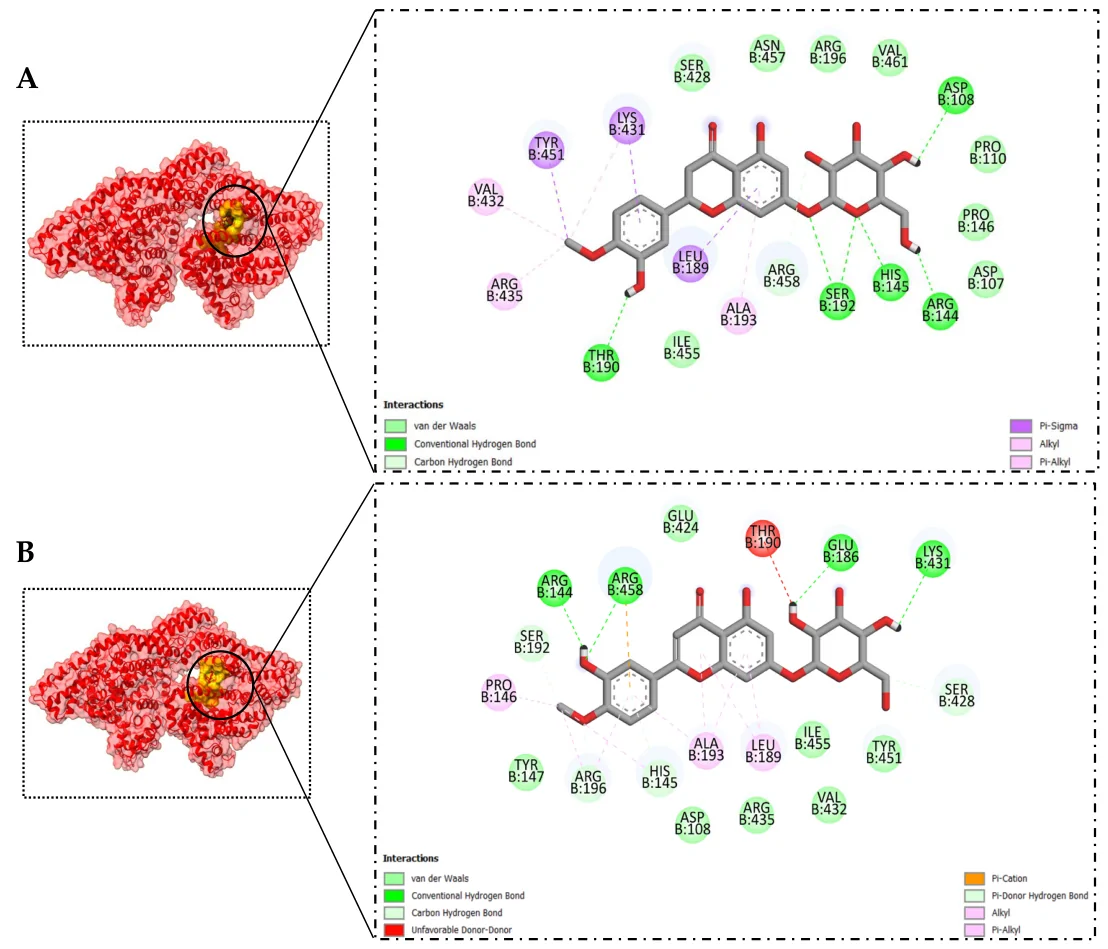
(**A**) Interactions between hesperetin-7-*O*-glucoside and BSA. (**B**) Interactions between diosmetin-7-*O*-glucoside and BSA.

The interaction between hesperetin-7-*O*-glucoside and bovine serum albumin (BSA) exhibited a binding free energy of −9.00 ± 0.091 kcal/mol. The main interactions involved hydrogen bonds with residues ARG B:144, ASP B:108, HIS B:145, and THR B:190, as well as π–sigma interactions with TYR B:451, LYS B:431, and LEU B:189 (Figure 5A).

The interaction between diosmetin-7-*O*-glucoside and bovine serum albumin (BSA) exhibited a binding free energy of −9.04 ± 0.059 kcal/mol. The main interactions involved hydrogen bonds with residues ARG B:144, ARG B:458, GLU B:186, and LYS B:431, as well as π–alkyl interactions with PRO B:146, ALA B:189, and LEU B:189. Van der Waals interactions with residue ARG B:196 were also observed (Figure 5B).

## 3. Discussion

Despite significant advances over the past decades in controlling hyperglycemia in diabetic patients—resulting from both lifestyle modifications and the development of more effective hypoglycemic drugs—chronic complications associated with diabetes mellitus remain a leading cause of morbidity and mortality worldwide. A large proportion of these complications is closely associated with increased oxidative stress, characterized by the excessive production of reactive oxygen species, and the activation of deleterious metabolic pathways, among which the formation of advanced glycation end products (AGEs) is particularly notable. In this context, the identification and development of bioactive compounds with antioxidant and antiglycation properties become crucial strategies for mitigating or preventing the molecular alterations involved in the onset of chronic diabetic complications.

In this regard, the results obtained in the present study demonstrate that the HCD and DCD formulations exhibit antioxidant activity across different chemical models. In the DPPH radical scavenging, total antioxidant capacity (TAC), and nitric oxide (NO) radical scavenging assays, both formulations showed measurable antioxidant effects, with concentration-dependent behavior observed in some of the evaluated assays, indicating their ability to interact with distinct reactive species and oxidative systems.

It is important to emphasize that the present study evaluated the HCD and DCD inclusion complex formulations as supplied by the manufacturer, rather than the isolated flavonoid glycosides. Cyclodextrins are primarily incorporated into these formulations to improve the physicochemical properties of poorly soluble compounds, including their solubility and stability. Consistent with this role, previous studies have reported that cyclodextrins alone did not exhibit DPPH radical scavenging activity and did not significantly affect browning product formation in a BSA-glucose glycation model under the evaluated experimental conditions [17,18]. However, because cyclodextrin-only control groups were not included in the present study and different experimental protocols were employed, a potential contribution of the excipients to the observed biological effects cannot be completely excluded. Therefore, the findings should be interpreted as the biological activity of the complete formulations rather than being attributed exclusively to the flavonoid glycosides.

In the evaluation of antioxidant potential using the DPPH method, which is based on the capacity of compounds to act as electron or hydrogen atom donors, both formulations exhibited a significant concentration-dependent response. Although the maximum DPPH scavenging values were moderate when compared with highly potent reference antioxidants, it should be noted that the tested concentrations were calculated based on the total mass of the formulations, which contain approximately 12–14% flavonoid glycosides and more than 50% cyclodextrins. Therefore, the effective concentrations of the flavonoid components at the highest tested formulation concentration were substantially lower than the nominal concentrations. Furthermore, the concentration range was selected to establish concentration–response relationships under controlled in vitro conditions and should not be interpreted as directly reflecting achievable systemic concentrations in vivo. These findings indicate that the formulations possess free radical scavenging activity, likely associated with the phenolic hydroxyl groups of the flavonoid moieties, which are known to stabilize radical species through electron or hydrogen-atom donation [19,20]. For the TAC and NO radical scavenging assays, DCD exhibited enhanced antioxidant capacity, which can be attributed to the structural characteristics of its aglycone (diosmetin). The presence of the C2=C3 double bond in the diosmetin structure confers higher planarity and electronic conjugation, promoting electron delocalization and, consequently, greater antioxidant efficiency, particularly in electron-transfer-based mechanisms [21,22]. The greater rigidity of the diosmetin structure may influence this improved biological activity.

In addition to the antioxidant activity demonstrated in chemical assays, the HCD and DCD formulations were evaluated in a cellular model of oxidative stress to determine whether the reactive species-neutralizing capacity observed in vitro would translate into protective effects in more complex biological systems. Two distinct models were employed: erythrocyte oxidative stress induced by AAPH and oxidative stress associated with high glucose concentrations, which mimics metabolically relevant conditions in the context of diabetes mellitus.

In the AAPH-induced model, a well-known thermal generator of peroxyl radicals, intense erythrocyte hemolysis was observed, highlighting the high oxidative potential of this system. Incubation of erythrocytes with the HCD and DCD formulations significantly reduced the percentage of hemolysis in a concentration-dependent manner, even under conditions of higher oxidative intensity. These results suggest that the active compounds present in the formulations, hesperetin-7-*O*-glucoside and diosmetin-7-*O*-glucoside, were capable of neutralizing peroxyl radicals before the onset of lipid peroxidation in the erythrocyte membrane, preserving its structural integrity. These findings are consistent with Liao et al. [23], who demonstrated that diosmetin attenuated AAPH-induced erythrocyte hemolysis and CuCl_2_-induced plasma oxidation by inhibiting intracellular reactive oxygen species production. Complementarily, Kim et al. [24] reported a significant reduction in hepatic lipid peroxidation in rats exposed to AAPH-induced oxidative stress and treated with hesperetin or hesperidin, reinforcing the protective potential of these flavonoids against oxidative damage. Thus, the results corroborate the chemical antioxidant assays and indicate that the radical-scavenging capacity translates into functional protection in cellular systems.

In contrast to the AAPH-induced oxidative stress model, high glucose concentrations trigger more complex mechanisms, involving increased reactive oxygen species production, depletion of endogenous antioxidant systems such as superoxide dismutase and catalase, as well as glutathione, and activation of glycation pathways [25]. In this context, the present study demonstrated that exposure of erythrocytes to high glucose concentrations induced significant alterations in cellular redox parameters, evidenced by increased levels of non-protein sulfhydryl groups and elevated catalase activity. These findings differ, at least in part, from those reported by Turpin et al. [26], who did not observe significant changes in catalase or superoxide dismutase activities in erythrocytes exposed to varying glucose concentrations.

The discrepancy between our findings and those reported by Turpin et al. [26] may be partially attributed to differences in incubation time, as prolonged glucose exposure is more likely to induce a progressive impairment of antioxidant defense systems as a consequence of sustained oxidative stress. However, additional experimental variables should also be taken into consideration, including the glucose concentration employed, the composition of the incubation medium, erythrocyte preparation procedures, and the specific redox biomarkers evaluated. Collectively, these factors may substantially influence the balance between adaptive antioxidant responses and oxidative cellular damage, thereby contributing to the divergent findings reported across different studies.

The observed increase in NPSH levels may be associated with metabolic adaptations induced by glucose exposure, including enhanced pentose phosphate pathway activity and increased NADPH generation, which support glutathione recycling and contribute to the maintenance of intracellular redox homeostasis. Additionally, considering the relatively short incubation period (5 h), the increase in NPSH may represent an early cellular response to glucose exposure rather than the depletion of thiol reserves typically observed during prolonged or chronic oxidative stress [27]. Nevertheless, since parameters directly related to glutathione metabolism, such as GSH, GSSG, or NADPH levels, were not evaluated in the present study, the precise biological significance of this finding remains to be further elucidated.

Furthermore, the increased catalase activity observed in the present study may represent a compensatory response to the elevated production of reactive oxygen species, particularly hydrogen peroxide, resulting from exposure to high glucose concentrations. This finding is consistent with Soares et al. [28], who reported a progressive increase in catalase and superoxide dismutase activities in erythrocytes incubated with gradually increasing glucose concentrations for 24 h, supporting the concept of an initial adaptive response of antioxidant defenses to hyperglycemic stress.

It is important to note that the erythrocyte model employed in the present study represents an acute exposure to high glucose rather than a chronic diabetic condition. Therefore, the observed alterations are more likely to reflect early redox adaptations and oxidative responses to hyperglycemia. Although this model is useful for investigating initial redox disturbances, longer exposure periods and in vivo diabetic models will be necessary to determine whether the protective effects of HCD and DCD are maintained under sustained hyperglycemic conditions.

Although the initial upregulation of antioxidant systems observed in the high-glucose model may represent an adaptive erythrocyte response to oxidative stress, this compensatory activation tends to be transient and insufficient under prolonged hyperglycemic stimulus. Extended exposure to elevated glucose levels promotes a progressive redox imbalance, characterized by depletion of endogenous antioxidant systems, accumulation of reactive oxygen species, and intensification of non-enzymatic glycation reactions [29]. This scenario favors the formation of highly reactive intermediates, such as methylglyoxal, culminating in the generation of advanced glycation end products (AGEs), protein oxidation, and structural and functional impairment of cells [30]. Thus, the adaptive response observed during the early phases of high glucose exposure can be interpreted as a temporary defense mechanism, whose failure over time directly contributes to the transition from compensated oxidative stress to chronic oxidative and glycative damage—a process closely associated with the development of late complications in diabetes mellitus [31].

Treatment with the formulations, particularly DCD, was able to modulate the adaptive alterations induced by high glucose. The reduction in non-protein sulfhydryl levels suggests decreased consumption of cellular antioxidants such as glutathione, while modulation of catalase activity indicates a possible restoration of intracellular redox homeostasis, or that hesperetin-7-*O*-glucoside may have acted as an enzymatic inducer. These effects support the hypothesis that the formulations act not only as direct scavengers of reactive species but also contribute to the preservation of endogenous antioxidant systems under metabolic stress. Furthermore, the antioxidant activity of the formulations under glycation-induced oxidative stress may be associated with their capacity to inhibit the formation of advanced glycation end products (AGEs), as previously demonstrated for various flavonoids [32].

The formation of advanced glycation end products (AGEs) is a consequence of the Maillard reaction, a series of non-enzymatic reactions between reducing sugars and amino groups of proteins, lipids, or nucleic acids. This reaction progresses from the formation of reversible adducts (Schiff bases) and Amadori products to the generation of stable AGEs, which can profoundly alter the structure and function of biomolecules. This process is widely recognized as a major contributor to the pathogenesis of diabetes-related diseases [33]. In this context, the antiglycation capacity of the HCD and DCD formulations was assessed using different in vitro glycation models to elucidate their possible mechanisms of action throughout the glycation process.

Initially, the bovine serum albumin (BSA)-fructose incubation model was employed, which is widely used to evaluate all stages of non-enzymatic glycation, as this reducing sugar exhibits high reactivity and promotes progression through all steps of the Maillard reaction [34]. In this model, the interaction between fructose and the free amino groups of BSA leads to the initial formation of Schiff bases, which evolve into Amadori products and subsequently into advanced glycation end products (AGEs), allowing an integrated analysis of the early, intermediate, and late stages of glycation, as well as protein oxidation. The quantification of fluorescent AGE formation in this system constitutes a sensitive approach to monitor the progression of protein glycation and the efficacy of antiglycation agents [35].

Thus, the reduction in fluorescence intensity observed for groups treated with the formulations in the present study indicates the ability of these compounds to broadly interfere with the glycation process, potentially through multiple mechanisms, including scavenging reactive carbonyl species, neutralizing reactive oxygen species involved in glycation amplification, and providing structural protection to proteins against oxidative modifications [12]. Moreover, the results regarding fructosamine levels, a marker of Amadori product formation, support the hypothesis that the formulations act not only by inhibiting the formation of advanced glycation end products but also during the early stages of the Maillard reaction, reinforcing their antiglycation potential across different phases of the glycation process. These findings are consistent with those reported by Ni et al. [13], who demonstrated that vitexin, a natural C-glycosylated flavonoid, was capable of inhibiting fluorescent AGE formation and reducing fructosamine levels, primarily through structural protection of BSA, methylglyoxal (MGO) scavenging, and modulation of glycation sites induced by protein interaction.

It is well established that the glycation process occurs concomitantly with oxidative stress, characterizing an integrated phenomenon known as glyco-oxidation. In this context, the degree of protein carbonylation and the oxidation of free thiol groups were evaluated in the fructose-induced BSA glycation model. Protein carbonylation is a key marker of oxidative damage, resulting both from the direct oxidation of amino acid side chains and from the reaction of proteins with reactive carbonyl species generated during glycation, such as methylglyoxal (MGO) [36]. Simultaneously, the reduction in free thiol groups reflects the oxidation of cysteine residues in BSA, which are highly sensitive to attack by reactive oxygen and carbonyl species. The loss of these groups compromises the structural integrity of the protein and diminishes its intrinsic antioxidant capacity, contributing to the progression of oxidative and glycative damage [37].

In this context, the results obtained demonstrated that, particularly, the DCD formulation was able to significantly attenuate protein glyco-oxidation, as evidenced by the reduction in protein carbonyl levels and the preservation of free thiol content. This effect may be associated with the consistent antioxidant activity of diosmetin-7-*O*-glycoside, which can act both by directly neutralizing reactive oxygen species and by scavenging highly reactive carbonyl intermediates. Furthermore, it is plausible that this flavonoid exerts an additional protective effect through interaction with susceptible protein residues, partially blocking glycation sites. These findings are in agreement with the study conducted by Khan et al. [38], in which the flavonoid naringenin was shown to significantly reduce protein carbonylation levels and attenuate the loss of free thiol groups in BSA subjected to methylglyoxal-induced glycation.

To evaluate the potential role of the formulations in scavenging carbonyl intermediates and protecting protein glycation sites, glycation assays were performed using bovine serum albumin (BSA) or arginine with methylglyoxal, a highly reactive dicarbonyl molecule. Methylglyoxal is considered one of the major precursors of advanced glycation end products (AGEs), exhibiting significantly higher reactivity than glucose or fructose, especially with arginine, lysine, and cysteine residues in proteins, rapidly leading to the formation of stable and structurally deleterious adducts [39]. In the context of diabetes mellitus, elevated MGO concentrations are observed as a consequence of increased glycolytic flux, triose phosphate fragmentation, reduced efficiency of the glyoxalase detoxification system, and glucose auto-oxidation, contributing decisively to carbonyl stress and to the exacerbation of chronic complications associated with hyperglycemia [8,40].

In this context, the HCD and DCD formulations significantly reduced the formation of fluorescent AGEs in both the bovine serum albumin (BSA) and arginine glycation models induced by methylglyoxal. However, a distinct behavior was observed between the formulations, suggesting action through partially different mechanisms. The DCD formulation exhibited more robust results in inhibiting AGE formation in the methylglyoxal-mediated glycation model, which aligns with the findings related to the reduction in protein carbonyl levels. These data suggest that DCD may more effectively interfere with glycation pathways involving reactive dicarbonyl species, such as methylglyoxal, thereby limiting the progression of glyco-oxidative reactions. Although direct methylglyoxal trapping was not evaluated in the present study, the superior activity observed in methylglyoxal-based models indicates a potential modulation of dicarbonyl-mediated glycation processes. These results are consistent with the findings of Li et al. [41], who demonstrated that the flavonoid quercetin was able to inhibit AGE formation through the direct scavenging of reactive dicarbonyls, such as methylglyoxal and glyoxal.

Conversely, the HCD formulation appears to exert its action predominantly through indirect antioxidant mechanisms and protein structural protection, possibly by preserving and/or blocking glycation-susceptible sites. This effect is supported by the results observed in the arginine model, as well as by the attenuation of free thiol oxidation, indicating a relevant role of HCD in maintaining the redox and structural integrity of proteins. These findings are in agreement with Li, Mitsuhashi, and Ubukata [42], who demonstrated the ability of hesperidin and its stereoisomers to trap reactive dicarbonyl species.

Another relevant strategy in inhibiting protein glycation is the capacity of certain natural compounds to bind competitively to proteins through non-covalent interactions, such as hydrogen bonds and van der Waals forces. These interactions can protect the structural integrity of proteins, reducing the accessibility of free amino groups and, consequently, inhibiting non-enzymatic glycation [4]. In this context, aiming to investigate the possible involvement of this mechanism in the experimentally observed effects, the in silico molecular interactions between hesperetin-7-*O*-glucoside and diosmetin-7-*O*-glucoside with bovine serum albumin (BSA) were evaluated. Both compounds exhibited considerable interaction energies with BSA, suggesting high binding affinity. Hesperetin-7-*O*-glucoside formed hydrogen bonds with the residues ARG B:144, ASP B:108, HIS B:145, and THR B:190, interactions that contribute significantly to the stability of the flavonoid–protein complex. Additionally, π–σ interactions involving the residues TYR B:451, LYS B:431, and LEU B:189 indicate complementary hydrophobic anchoring, favoring the ligand’s persistence at the protein interface. Notably, the flavonoid is positioned near residue ARG B:196, an amino acid recognized as involved in the glycation process [43]. Similarly, diosmetin-7-*O*-glucoside exhibited a robust interaction profile with BSA, forming hydrogen bonds with the residues ARG B:144, ARG B:458, GLU B:186, and LYS B:431. Furthermore, the presence of van der Waals interactions with residue ARG B:196 supports the hypothesis that diosmetin-7-*O*-glucoside also contributes to the direct protection of critical regions of BSA against glycation modification [43,44]. These findings provide supportive mechanistic evidence that the formulations may exert additional antiglycation effects through protein structural stabilization and steric blocking of glycation-susceptible sites, potentially contributing to the reduction in glycation product formation observed experimentally.

Taken together, the glycation results indicate that the HCD and DCD formulations act at multiple levels of the glycation process, interfering both with early-stage reactions (fructosamine formation) and the progression toward AGEs, as well as secondary oxidative protein damage. The DCD formulation, in particular, demonstrated a more pronounced antiglycation profile among the tested formulations, with effects comparable to aminoguanidine in selected parameters, such as fructosamine formation and protein carbonylation. These findings support further investigation into the mechanisms underlying the antiglycation activity of DCD and its potential relevance in modulating molecular alterations associated with hyperglycemia.

Although the present study provides consistent evidence of the antioxidant and antiglycation activities of the HCD and DCD formulations using complementary in vitro, ex vivo, and in silico approaches, several limitations should be acknowledged. The experimental models employed cannot fully reproduce the complexity of the diabetic microenvironment, including factors such as metabolism, tissue distribution, pharmacokinetics, bioavailability, and long-term biological responses. Likewise, the molecular docking analysis provides only a theoretical prediction of ligand–protein interactions and should be interpreted as supportive computational evidence rather than experimental confirmation of the proposed mechanisms. Therefore, further pharmacokinetic, mechanistic, and in vivo studies will be essential to establish the translational relevance and therapeutic potential of these formulations.

## 4. Materials and Methods

### 4.1. Reagents and Compounds

The formulations hesperetin-7-*O*-glucoside complexed with β-cyclodextrin (SunActive^®^, 14.2–14.5% HEPT7G and 57.5% β-CD) and diosmetin-7-*O*-glucoside complexed with γ-cyclodextrin (SunActive^®^, 12.0–12.3% DIOSG and 51% γ-CD) are manufactured by Taiyo Kagaku Co., Ltd. (Yokkaichi, Japan). The concentrations used throughout the study were calculated based on the total mass of the formulations. The structural characterization of the hesperetin-7-*O*-glucoside/β-cyclodextrin inclusion complex has been reported by Kapoor et al. [45], while the physicochemical and biopharmaceutical properties of these formulations have been described by Moriwaki et al. [46,47]. Bovine serum albumin (BSA), methylglyoxal (MGO), nitroblue tetrazolium (NBT), 2,2-diphenyl-1-picrylhydrazyl (DPPH), sodium nitroprusside (SNP), 2,2′-azobis(2-amidinopropane) dihydrochloride (AAPH), 5,5′-dithiobis-(2-nitrobenzoic acid) (DTNB), and 2,4-dinitrophenylhydrazine (DNPH) were purchased from commercial suppliers at analytical grade (Sigma-Aldrich, St. Louis, MO, USA). All solutions were prepared in appropriate buffers immediately before use.

### 4.2. Antioxidant Activity in Chemical Models

#### 4.2.1. Determination of DPPH Radical Scavenging Activity

The DPPH (2,2-diphenyl-1-picrylhydrazyl) radical-scavenging activity was performed according to Rufino et al. [48]. Aliquots of 200 μL of HCD and DCD (100–1600 μg/mL, in PBS 0.111 M, pH 7.4) were added to 2.0 mL of methanolic DPPH solution. The mixtures were incubated for 60 min in the dark, and the decrease in absorbance was measured at 517 nm (Bioespectro SP220, Biospectro, Curitiba, Brazil). The negative control consisted of methanol + DPPH, and the blank contained PBS + methanol. Quercetin (200 μg/mL) was used as the positive control. The scavenging activity was expressed as the percentage of inhibition relative to the control, calculated using the following formula: %A = [(Abs_DPPH_ − (Abs_sample_ − Abs_blank_)/Abs_DPPH_)] × 100.

#### 4.2.2. Total Antioxidant Capacity (TAC) Quantification

The total antioxidant capacity was determined using the phosphomolybdenum method, as described by Prieto et al. [49]. Aliquots of 200 μL of HCD and DCD (100–1600 μg/mL, in PBS 0.111 M, pH 7.4) were added to 2.0 mL of ammonium phosphomolybdate reagent. The mixtures were homogenized and incubated at 37 °C for 30 min. The blank was prepared by replacing the sample with an equal volume of PBS. After incubation, the absorbance was measured at 695 nm using a spectrophotometer (Bioespectro SP220, Biospectro, Curitiba, Paraná, Brazil). Results were expressed as ascorbic acid equivalents, calculated from a calibration curve obtained using ascorbic acid as the standard.

#### 4.2.3. Nitric Oxide (NO) Radical Scavenging Assay

The nitric oxide scavenging activity was assessed based on the spontaneous release of NO from sodium nitroprusside in aqueous solution (pH 7.4), as described by Kurian et al. [50]. The generated NO reacts with molecular oxygen to form nitrite, which was quantified using Griess reagent (Merck KGaA, Darmstadt, Germany). Aliquots of 200 µL of sodium nitroprusside (10 mM, in sodium phosphate buffer, pH 7.4) were incubated with 200 µL of HCD or DCD (100–1600 µg/mL) at 37 °C for 2 h. After incubation, 200 µL of Griess reagent was added, and the absorbance was measured at 546 nm using a microplate reader (Glomax^®^ Discover, Promega, Madison, WI, USA). The negative control consisted of sodium nitroprusside in the absence of formulations, and Trolox (750 µg/mL) was used as a positive control. Results were expressed as the percentage inhibition of nitrite formation relative to the control, calculated by the equation: %A = (1 − Abs_sample_/Abs_negative control_) × 100.

### 4.3. Antioxidant Activity in a Cellular Model of Oxidative Stress

#### 4.3.1. Animals and Erythrocyte Collection

Thirteen male Wistar rats (*Rattus norvegicus*), weighing between 230 and 270 g and aged 6 to 8 months, were obtained from the Central Bioterium of the Federal University of Piauí (UFPI). The animals were maintained under controlled conditions of temperature (25 ± 2 °C), with a 12 h light/12 h dark photoperiod, and had ad libitum access to standard chow and water. All experimental procedures were approved by the UFPI Ethics Committee on Animal Use (CEUA-UFPI; approval no. 833/2024) and conducted in accordance with the guidelines of the National Council for the Control of Animal Experimentation (CONCEA).

Euthanasia was performed by an overdose of thiopental (150 mg/kg, intraperitoneally). Blood was collected via puncture of the hepatic portal vein into tubes containing EDTA. After centrifugation, plasma and the leukocyte layer were removed, and erythrocytes were washed three times with phosphate-buffered saline (PBS, 300 mOsm, pH 7.4). The washed erythrocyte suspension was subsequently used in oxidative stress assays induced either by AAPH or by high glucose concentrations.

#### 4.3.2. Anti-Hemolytic Activity (AAPH-Induced Oxidative Stress)

The anti-hemolytic activity was assessed according to the method of Yang et al. [51], with modifications. Washed erythrocyte suspensions were adjusted to 10% hematocrit and pre-incubated with HCD or DCD (25–200 μg/mL) for 60 min at 37 °C. Following pre-treatment, AAPH solution was added to achieve final concentrations of 35 or 50 mM, and samples were incubated for 2 h at 37 °C to induce oxidative hemolysis. After incubation, the final volume was adjusted to 1.8 mL with PBS (0.111 M, pH 7.4), and samples were centrifuged at 2500 rpm for 10 min. The absorbance of the supernatant was measured at 540 nm using a microplate reader (Glomax^®^ Discover, Promega, Madison, WI, USA), with hemoglobin release serving as an indicator of hemolysis. Trolox (375 μg/mL) was used as a positive control, while 1% Triton X-100 was employed to induce 100% hemolysis. The percentage of hemolysis was calculated using the equation: %hemolysis = 100 − {[(Abs_triton X-100_ − Abs_sample_)/(Abs_triton-X_ 1% − Abs_blank_)] × 100} where Abs_triton-X_ is the absorbance of erythrocytes incubated with 1% Triton X-100, Abs_sample_ is the absorbance of erythrocytes pre-treated with the test formulation or vehicle and subjected to AAPH-induced hemolysis and Abs_blank_ is the absorbance of erythrocytes pre-treated with vehicle and not subjected to hemolysis.

#### 4.3.3. High Glucose-Induced Oxidative Stress Model

The erythrocyte concentrate was incubated for 5 h at 37 °C in a medium composed of PBS, 1% bovine serum albumin (BSA), 2% glucose, and different concentrations of the formulations (DCD and HCD at 200, 400, and 800 µg/mL). The blank group was incubated in the same medium without glucose, while aminoguanidine (50 µM) was used as a positive control. Aliquot blood samples from each animal, our experimental unit, were used for all groups. At the end of the incubation period, erythrocytes were washed again with PBS and aliquoted for subsequent analyses [52,53,54].

#### Determination of Non-Protein Sulfhydryl Groups (NPSH)

Non-protein sulfhydryl groups were quantified according to Giustarini et al. [55], with modifications. After oxidative stress induction, 60 μL of erythrocyte suspension was added to 1.8 mL of stabilizing solution containing EDTA and EGTA (450 μM). Protein precipitation was performed by adding 60% (*w*/*v*) TCA, followed by centrifugation at 5000 rpm for 5 min. The supernatant was transferred to a new tube, and the pH was adjusted with 2 M TRIS. Subsequently, 3 mM DTNB solution (in 0.2 M potassium phosphate buffer, pH 6.2) was added to reach a final concentration of 10 μM. The formation of the chromogenic complex was monitored at 412 nm using a spectrophotometer (Bioespectro SP220, Biospectro, Curitiba, Brazil). NPSH concentrations were determined by linear correlation from a calibration curve constructed with standard cysteine solutions and expressed in µmol/L.

#### Assessment of Erythrocyte Catalase Activity

Catalase activity was determined according to Beutler [56], based on the decomposition of hydrogen peroxide (H_2_O_2_), monitored by the decrease in absorbance at 230 nm at 37 °C. For the assay, 10 μL of erythrocyte lysate was added to 2.0 mL of reaction medium containing phosphate buffer (50 mM, pH 7.0) and H_2_O_2_. The change in optical density was recorded at 0 and 30 s using a spectrophotometer (Bioespectro SP220, Biospectro, Curitiba, Brazil). Enzymatic activity was expressed as the rate of absorbance change per minute, normalized to hemoglobin concentration (determined using the Drabkin reagent, Labtest, Lagoa Santa, Minas Gerais, Brazil).

### 4.4. In Vitro Evaluation of Antiglycation Activity

#### 4.4.1. Bovine Serum Albumin (BSA) Glycation Model Induced by Fructose (FRU)

Bovine serum albumin (BSA) was subjected to in vitro glycation by fructose (FRU) following the protocol described by Franco et al. [57], with minor adaptations. Briefly, a reaction mixture was prepared containing BSA (50 mg/mL), FRU (1.25 M) in sodium phosphate buffer (0.111 M, pH 7.4), and serial concentrations of the formulations (HCD and DCD at 100, 200, and 400 µg/mL). Incubation was carried out under sterile conditions at 37 °C, in the dark, for 7 days. Aminoguanidine (550 µg/mL) was used as a positive control.

After incubation, samples were diluted 1:5 (*v*/*v*) in PBS (0.111 M, pH 7.4), and fluorescence intensity was measured using a Glomax^®^ Discover Microplate Reader (Promega Corporation, Madison, WI, USA) at excitation/emission wavelengths of 355/460 nm. The percentage of protein glycation inhibition was calculated using the following equation: %inhibition= [1 − (Fsample − Fblank1/Fcontrol − Fblank2)] × 100, where Fcontrol is the fluorescence intensity of fructose-mediated AGEs without sample; Fsample is the fluorescence intensity of fructose-mediated AGEs treated with the formulations or positive control; Fblank1is the fluorescence of the BSA–sample mixture without fructose; and Fblank2 is the fluorescence of BSA alone (without fructose or sample).

#### Determination of Fructosamine Levels

Fructosamine levels were determined according to Baker et al. [58], based on the reduction in nitroblue tetrazolium (NBT). Glycated and non-glycated samples were incubated with NBT (250 μM) in carbonate buffer (0.1 M, pH 10.4) at 37 °C for 2 h. After incubation, absorbance was measured at 525 nm using a microplate reader (Glomax^®^ Discover, Promega, Madison, WI, USA). Quantification was performed using a molar extinction coefficient of 12,640 M^−1^·cm^−1^ for monoformazan. The percentage of inhibition of fructosamine formation was calculated using the following equation: %inhibition = [1 − (Abs_sample_ − Abs_blank_/Abs_control_ − Abs_blank_)] × 100, where Abs_sample_ is the absorbance of the glycated samples treated with the formulations or positive control, Abs_blank_ is the absorbance of non-glycated BSA, and Abs_control_ is the absorbance of glycated BSA without treatment.

#### Determination of Protein Carbonyl Content

Protein carbonyl content was determined as described by Levine et al. [59], with modifications. Aliquots of glycated and non-glycated samples were incubated with 2,4-dinitrophenylhydrazine (DNPH, 10 mM in 2.5 M HCl) for 60 min at room temperature, protected from light. The reaction was stopped by adding trichloroacetic acid (20%, *w*/*v*), followed by incubation on ice and centrifugation to precipitate proteins. The resulting pellet was washed with an ethanol/ethyl acetate solution (1:1, *v*/*v*) to remove excess reagents and subsequently solubilized in 6 M guanidine hydrochloride. Absorbance was measured at 370 nm using a spectrophotometer (Bioespectro SP220, Biospectro, Curitiba, Paraná, Brazil). Carbonyl concentration was calculated using a molar extinction coefficient of 22,000 M^−1^·cm^−1^ for the formed hydrazones. The percentage of inhibition of protein carbonyl formation was determined using the same equation described in Section Determination of Fructosamine Levels.

#### Determination of Free Thiol Group Concentration

Free thiol groups were determined according to Ellman [60], using 5,5′-dithiobis-(2-nitrobenzoic acid) (DTNB). Aliquots of glycated and non-glycated samples were incubated with DTNB (6 mM in 0.1 M PBS, pH 7.4) for 15 min at room temperature, protected from light. After incubation, absorbance was measured at 412 nm using a spectrophotometer (Bioespectro SP220, Biospectro, Curitiba, Paraná, Brazil). Thiol concentration was calculated using the molar extinction coefficient of 5-thio-2-nitrobenzoate (TNB) (ε = 13,600 M^−1^·cm^−1^), and results were expressed as nmol of free thiols per mg of protein.

#### 4.4.2. Bovine Serum Albumin (BSA) Glycation Model Induced by Methylglyoxal (MGO)

Bovine serum albumin (BSA) was subjected to in vitro glycation induced by methylglyoxal (MGO), following the protocol described by Khan et al. [38], with minor modifications. The reaction mixture contained BSA (10 mg/mL), MGO (6 mM) in sodium phosphate buffer (0.111 M, pH 7.4), and serial concentrations of the formulations (HCD and DCD at 100, 200, and 400 µg/mL). Incubation was performed under sterile conditions at 37 °C, in the dark, for 7 days. Aminoguanidine (275 µg/mL) was used as a positive control. After incubation, samples were diluted 1:5 (*v*/*v*) in PBS (0.111 M, pH 7.4), and fluorescence intensity was measured using a Glomax^®^ Discover Microplate Reader (Promega Corporation, Madison, WI, USA) at excitation/emission wavelengths of 355/460 nm. The percentage of glycation inhibition was calculated using the same equation described for the BSA–fructose model.

#### 4.4.3. Arginine (ARG) Glycation Model Induced by Methylglyoxal (MGO)

Arginine (ARG) was subjected to in vitro glycation induced by methylglyoxal (MGO), following the protocol described by Justino et al. [61], with minor modifications. The reaction mixture consisted of ARG (22 mM), MGO (6 mM) in sodium phosphate buffer (0.111 M, pH 7.4), and serial concentrations of the formulations (HCD and DCD at 100, 200, and 400 µg/mL). Incubation was performed under sterile conditions at 37 °C, in the dark, for 4 days. Aminoguanidine (550 µg/mL) was used as a positive control. After incubation, samples were diluted 1:5 (*v*/*v*) in PBS (0.111 M, pH 7.4), and fluorescence intensity was measured using a Glomax^®^ Discover Microplate Reader (Promega Corporation, Madison, WI, USA) at excitation/emission wavelengths of 355/460 nm. The percentage of glycation inhibition was calculated using the same equation described for the BSA–fructose model.

#### 4.4.4. Molecular Docking

Molecular docking was performed to evaluate the interaction of hesperetin-7-*O*-glucoside (HCD) and diosmetin-7-*O*-glucoside (DCD) with bovine serum albumin (BSA). The three-dimensional structure of the protein was obtained from the Protein Data Bank (PDB ID: 3V03), while the 3D structures of the ligands were retrieved from the PubChem database. Protein preparation involved the removal of water molecules and cofactors, followed by the addition of polar hydrogens and assignment of Gasteiger charges. The binding site was defined based on cavity detection using Molegro Virtual Docker 6.0 (Molegro, A CLC bio company, Aarhus, Denmark) software, considering the grid box center coordinates (X = 65.11; Y = 25.79; Z = 28.17) and dimensions of 60 × 60 × 60 Å. Docking simulations were carried out using AutoDock Vina 1.2.3 after structure preparation in AutoDock Tools (v1.5.7) (Scripps Research Institute, La Jolla, CA, USA). Binding free energy (ΔG) values were used as the affinity parameter, and ligand–amino acid residue interactions were analyzed. Two- and three-dimensional representations of the best-ranked poses were generated using Discovery Studio Visualizer 2021 (Dassault Systèmes BIOVIA, San Diego, CA, USA) and ChimeraX 1.9 (RBVI, University of California, San Francisco, CA, USA) software, enabling the identification of hydrogen bonds, electrostatic interactions, and van der Waals forces [62,63,64].

### 4.5. Statistical Analysis

Statistical analyses were performed using GraphPad Prism software (version 8.0.2; GraphPad Software Inc., San Diego, CA, USA). For cell-free assays, including the chemical antioxidant assay and BSA glycation models, each experimental condition was analyzed in technical triplicate. For erythrocyte-based assays, independent biological replicates were obtained using erythrocytes collected from 3 to 5 animals, depending on the assay. Data are expressed as mean ± standard error of the mean (SEM). Statistical significance was determined using one-way and two-way analysis of variance (ANOVA), followed by Dunnett’s or Tukey’s post hoc tests for multiple comparisons, as appropriate. As data was normally distributed (Shapiro–Wilk’s test) and homoscedastic (Bartlett’s test). Differences were considered statistically significant when *p* < 0.05; no outliers were found, and no experimental unit was excluded from the results.

## 5. Conclusions

The results demonstrate that the HCD and DCD formulations exhibit consistent antioxidant activity across different experimental protocols, effectively inhibiting the formation of radical species involved in oxidative stress. Furthermore, the evaluated formulations displayed promising antiglycation activity. While the HCD formulation showed a profile consistent with indirect antioxidant effects and protein protection, the DCD formulation demonstrated greater efficacy in experimental models involving dicarbonyl-dependent glycation pathways, suggesting potential differences in the mechanisms underlying their biological activities. Further mechanistic investigations will be necessary to confirm these observations.

Moreover, the HCD and DCD formulations exhibited antioxidant and antiglycation activities that may help attenuate molecular alterations associated with chronic hyperglycemia, including oxidative stress and non-enzymatic protein glycation. However, additional in vivo studies and complementary mechanistic investigations are required to confirm these effects and further elucidate their potential pharmacological relevance.

## Data Availability

The original contributions presented in the study are included in the article, further inquiries can be directed to the corresponding author.

## Abbreviations

3-DG, 3-deoxyglucosone; AAPH, 2,2′-azobis(2-methylpropionamidine) dihydrochloride; ABS, absorbance; AGEs, advanced glycation end products; ARG, arginine; BSA, bovine serum albumin; CAOT, total antioxidant capacity; CAT, catalase; CDs, cyclodextrins; CEUA, Ethics Committee on Animal Use; CONCEA, National Council for the Control of Animal Experimentation; DCD, diosmetin-7-*O*-glucoside complexed with γ-cyclodextrin; DM, diabetes mellitus; DM1, type 1 diabetes mellitus; DM2, type 2 diabetes mellitus; DNPH, 2,4-dinitrophenylhydrazine; DPPH, 2,2-diphenyl-1-picrylhydrazyl; DTNB, 5,5′-dithiobis-(2-nitrobenzoic acid); EDTA, ethylenediaminetetraacetic acid; EGTA, ethylene glycol-bis(β-aminoethyl ether)-N,N,N′,N′-tetraacetic acid; ERNs (RNS), reactive nitrogen species; EROs (ROS), reactive oxygen species; FRU, fructose; GO, glyoxal; GPx, glutathione peroxidase; GSNP (NP-SH), non-protein sulfhydryl groups; H_2_O_2_, hydrogen peroxide; HbA1c, glycated hemoglobin; HCD, hesperetin-7-*O*-glucoside complexed with β-cyclodextrin; HCl, hydrochloric acid; IDF, International Diabetes Federation; IL-1, interleukin-1; IL-6, interleukin-6; MGO, methylglyoxal; NBT, nitroblue tetrazolium; NF-κB, nuclear factor kappa B; NO, nitric oxide; O_2_^−^, superoxide anion; ONOO^−^, peroxynitrite; PBS, sodium phosphate buffer (0.111 M, pH 7.4); PDB, Protein Data Bank; PKC, protein kinase C; RAGE, receptor for advanced glycation end products; SOD, superoxide dismutase; TCA, trichloroacetic acid; TNF-α, tumor necrosis factor alpha; TOTG (OGTT), oral glucose tolerance test; UFPI, Federal University of Piauí; VCAM-1, vascular cell adhesion molecule-1; β-CD, beta-cyclodextrin; γ-CD, gamma-cyclodextrin.

## References

[1] International Diabetes Federation (2025). IDF Diabetes Atlas.

[2] Rodacki M., Teles M., Gabbay M., Montenegro R., Bertoluci M., Lamounier R. (2023). Classificação do diabetes. Diretriz Oficial da Sociedade Brasileira de Diabetes.

[3] Ahmad S., Khan M.S., Akhter F., Khan M.S., Khan A., Ashraf J.M., Pandey R.P., Shahab U. (2014). Glycoxidation of Biological Macromolecules: A Critical Approach to Halt the Menace of Glycation. Glycobiology.

[4] Song Q., Liu J., Dong L., Wang X., Zhang X. (2021). Novel advances in inhibiting advanced glycation end product formation using natural compounds. Biomed. Pharmacother..

[5] Yaribeygi H., Sathyapalan T., Atkin S.L., Sahebkar A. (2020). Molecular Mechanisms Linking Oxidative Stress and Diabetes Mellitus. Oxid. Med. Cell. Longev..

[6] Bhatti J.S., Sehrawat A., Mishra J., Sidhu I.S., Navik U., Khullar N., Kumar S., Bhatti G.K., Reddy P.H. (2022). Oxidative stress in the pathophysiology of type 2 diabetes and related complications: Current therapeutics strategies and future perspectives. Free Radic. Biol. Med..

[7] Maessen D.E.M., Stehouwer C.D.A., Schalkwijk C.G. (2015). The role of methylglyoxal and the glyoxalase system in diabetes and other age-related diseases. Clin. Sci..

[8] Rabbani N., Thornalley P.J. (2014). The critical role of methylglyoxal and glyoxalase 1 in diabetic nephropathy. Diabetes.

[9] Nowotny K., Jung T., Höhn A., Weber D., Grune T. (2015). Advanced glycation end products and oxidative stress in type 2 diabetes mellitus. Biomolecules.

[10] Zhou M., Zhang Y., Shi L., Li L., Zhang D., Gong Z., Wu Q. (2024). Activation and modulation of the AGEs-RAGE axis: Implications for inflammatory pathologies and therapeutic interventions—A review. Pharmacol. Res..

[11] Ramis R., Casasnovas R., Mariño L., Frau J., Adrover M., Vilanova B., Mora-Diez N., Ortega-Castro J. (2019). A density functional theory study of the free-radical scavenging activity of aminoguanidine. Comparison with its reactive carbonyl compound and metal scavenging activities. Int. J. Quantum Chem..

[12] Yeh W.-J., Hsia S.-M., Lee W.-H., Wu C.-H. (2017). Polyphenols with antiglycation activity and mechanisms of action: A review of recent findings. J. Food Drug Anal..

[13] Ni M., Song X., Pan J., Gong D., Zhang G. (2021). Vitexin inhibits protein glycation through structural protection, methylglyoxal trapping, and alteration of glycation site. J. Agric. Food Chem..

[14] Karak P. (2019). Biological activities of flavonoids: An overview. Int. J. Pharm. Sci. Res..

[15] Jansook P., Ogawa N., Loftsson T. (2018). Cyclodextrins: Structure, physicochemical properties and pharmaceutical applications. Int. J. Pharm..

[16] Kfoury M., Fourmentin S. (2024). State of the art in cyclodextrin solubility enhancement. Are green solvents the solution?. J. Mol. Liq..

[17] Lu S., Cheng B., Hu Y., Zhang Y., Zou G. (2009). Complexation of resveratrol with cyclodextrins: Solubility and antioxidant activity. Food Chem..

[18] Favre L.C., Santos C., López-Fernández M.P., Mazzobre M.F., Buera M.P. (2018). Optimization of β-cyclodextrin-based extraction of antioxidant and anti-browning activities from thyme leaves by response surface methodology. Food Chem..

[19] Platzer M., Kiese S., Tybussek T., Herfellner T., Schneider F., Schweiggert-Weisz U., Eisner P. (2022). Radical scavenging mechanisms of phenolic compounds: A quantitative structure–property relationship (QSPR) study. Front. Nutr..

[20] Choi S.S., Lee S.H., Lee K.A. (2022). A comparative study of hesperetin, hesperidin and hesperidin glucoside: Antioxidant, anti-inflammatory, and antibacterial activities in vitro. Antioxidants.

[21] Hassanpour S.H., Doroudi A. (2023). Review of the antioxidant potential of flavonoids as a subgroup of polyphenols and partial substitute for synthetic antioxidants. Avicenna J. Phytomed..

[22] Lee H.J., Lee S.H., Hong S.K., Gil B.I., Lee K.A. (2024). In vitro biological activities of hesperidin-related compounds with different solubility. Antioxidants.

[23] Liao W., Ning Z., Chen L., Wei Q., Yuan E., Yang J., Ren J. (2014). Intracellular antioxidant detoxifying effects of diosmetin on 2,2-azobis(2-amidinopropane) dihydrochloride (AAPH)-induced oxidative stress through inhibition of reactive oxygen species generation. J. Agric. Food Chem..

[24] Kim J.H., Li L., Mi S., Eun J.C., Hyun Y.K., Jine S.C. (2022). Hesperidin and hesperetin protect against oxidative stress on hepatic toxicity in rats. J. Korean Med. Obes. Res..

[25] Obeagu E.I. (2024). Red blood cells as biomarkers and mediators in complications of diabetes mellitus: A review. Medicine.

[26] Turpin C., Catan A., Guerin-Dubourg A., Debussche X., Bravo S.B., Álvarez E., Van Den Elsen J., Meilhac O., Rondeau P., Bourdon E. (2020). Enhanced oxidative stress and damage in glycated erythrocytes. PLoS ONE.

[27] Rogers S.C., Ross J.G., D’Avignon A., Gibbons L.B., Gazit V., Hassan M.N., McLaughlin D., Griffin S., Neumayr T., DeBaun M. (2013). Sickle hemoglobin disturbs normal coupling among erythrocyte O_2_ content, glycolysis, and antioxidant capacity. Blood.

[28] Soares J.C.M., Folmer V., da Rocha J.B.T., Nogueira C.W. (2014). Ebselen exhibits glycation-inhibiting properties and protects against osmotic fragility of human erythrocytes in vitro. Cell Biol. Int..

[29] Papachristoforou E., Lambadiari V., Maratou E., Makrilakis K. (2020). Association of glycemic indices (hyperglycemia, glucose variability, and hypoglycemia) with oxidative stress and diabetic complications. J. Diabetes Res..

[30] Khanam A., Ahmad S., Husain A., Rehman S., Farooqui A., Yusuf M.A. (2020). Glycation and antioxidants: Hand in the glove of antiglycation and natural antioxidants. Curr. Protein Pept. Sci..

[31] Albert-Garay J.S., Riesgo-Escovar J.R., Salceda R. (2022). High glucose concentrations induce oxidative stress by inhibiting Nrf2 expression in rat Müller retinal cells in vitro. Sci. Rep..

[32] Li L.-F., Wang M.-D., Zhang C.-Y., Jin M.-Y., Chen H.-L., Luo H., Hou T.-Y., Zhang Z.-J., Li H. (2025). Influence of hydroxyl substitution on the inhibition of flavonoids in advanced glycation end-products formation in glucose-lysine-arginine Maillard reaction models. Food Res. Int..

[33] Shen C.Y., Lu C.H., Wu C.H., Li K.J., Kuo Y.M., Hsieh S.C., Yu C.L. (2020). The development of Maillard reaction, and advanced glycation end product (AGE)-receptor for AGE (RAGE) signaling inhibitors as novel therapeutic strategies for patients with AGE-related diseases. Molecules.

[34] Muraoka M.Y., Justino A.B., Caixeta D.C., Queiroz J.S., Sabino-Silva R., Salmen Espindola F. (2022). Fructose and methylglyoxal-induced glycation alters structural and functional properties of salivary proteins, albumin and lysozyme. PLoS ONE.

[35] Sadowska-Bartosz I., Galiniak S., Bartosz G. (2014). Kinetics of glycoxidation of bovine serum albumin by glucose, fructose and ribose and its prevention by food components. Molecules.

[36] Martínez-Orgado J., Martínez-Vega M., Silva L., Romero A., De Hoz-Rivera M., Villa M., Del Pozo A. (2023). Protein carbonylation as a biomarker of oxidative stress and a therapeutic target in neonatal brain damage. Antioxidants.

[37] Acimovic J., Stanimirovic B., Mandic L. (2009). The role of the thiol group in protein modification with methylglyoxal. J. Serbian Chem. Soc..

[38] Khan M.S., Qais F.A., Rehman M.T., Ismail M.H., Alokail M.S., Altwaijry N., Alafaleq N.O., AlAjmi M.F., Salem N., Alqhatani R. (2020). Mechanistic inhibition of non-enzymatic glycation and aldose reductase activity by naringenin: Binding, enzyme kinetics and molecular docking analysis. Int. J. Biol. Macromol..

[39] Vašková J., Kováčová G., Pudelský J., Palenčár D., Mičková H. (2025). Methylglyoxal formation—Metabolic routes and consequences. Antioxidants.

[40] Schalkwijk C.G., Stehouwer C.D.A. (2020). Methylglyoxal, a highly reactive dicarbonyl compound, in diabetes, its vascular complications, and other age-related diseases. Physiol. Rev..

[41] Li X., Zheng T., Sang S., Lv L. (2014). Quercetin inhibits advanced glycation end product formation by trapping methylglyoxal and glyoxal. J. Agric. Food Chem..

[42] Li D., Mitsuhashi S., Ubukata M. (2012). Protective effects of hesperidin derivatives and their stereoisomers against advanced glycation end-products formation. Pharm. Biol..

[43] Abbasi S., Gharaghani S., Benvidi A., Rezaeinasab M. (2018). New insights into the efficiency of thymol synergistic effect with p-cymene in inhibiting advanced glycation end products: A multi-way analysis based on spectroscopic and electrochemical methods in combination with molecular docking study. J. Pharm. Biomed. Anal..

[44] Hinton D.J.S., Ames J.M. (2007). Site specificity of glycation and carboxymethylation of bovine serum albumin by fructose. Amino Acids.

[45] Kapoor M.P., Moriwaki M., Minoura K., Timm D., Abe A., Kito K. (2022). Structural Investigation of Hesperetin-7-*O*-Glucoside Inclusion Complex with β-Cyclodextrin: A Spectroscopic Assessment. Molecules.

[46] Moriyaki M., Kito K., Nakagawa R., Kapoor M.P., Matsumiya Y., Fukuhara T., Kamiya U. (2021). Bioavailability comparison between a compound comprising hesperetin-7-glucoside with β-cyclodextrin and a mixture of hesperidin and dextrin in healthy adult human males. Biosci. Biotechnol. Biochem..

[47] Moriwaki M., Kito K., Nakagawa R., Kapoor M.P., Matsumiya Y., Fukuhara T., Kobayashi J., Satomoto K., Yamagata H., Kuroiwa Y. (2023). Increased bioavailability of diosmetin-7-glucoside-γ-cyclodextrin inclusion complex compared with diosmin in Sprague-Dawley rats. Biosci. Biotechnol. Biochem..

[48] Rufino M.S.M., Alves R.E., Brito E.S., Morais S.M., Sampaio C.G., Pérez-Jiménez J., Saura-Calixto F.D. (2007). Metodologia científica: Determinação da atividade antioxidante total em frutas pela captura do radical livre DPPH. Embrapa Agroindústria Tropical, Comunicado Técnico.

[49] Prieto P., Pineda M., Aguilar M. (1999). Spectrophotometric quantitation of antioxidant capacity through the formation of a phosphomolybdenum complex: Application to the determination of vitamin E. Anal. Biochem..

[50] Kurian V., Patil L., Pathak A., Chandra N. (2010). Free radicals, antioxidants and functional foods: Impact on human health. Pharmacogn. Rev..

[51] Yang H.L., Korivi M., Lin M.K., Chang H.C.W., Wu C.R., Lee M.S., Hseu Y.C. (2017). Antihemolytic and antioxidant properties of pearl powder against 2,2′-azobis(2-amidinopropane) dihydrochloride-induced hemolysis and oxidative damage to erythrocyte membrane lipids and proteins. J. Food Drug Anal..

[52] Hou F.F., Boyce J., Chertow G.M., Kay J., Owen W.F. (1998). Aminoguanidine inhibits advanced glycation end products formation on beta2-microglobulin. J. Am. Soc. Nephrol..

[53] Thornalley P.J. (2003). Use of aminoguanidine (Pimagedine) to prevent the formation of advanced glycation end products. Arch. Biochem. Biophys..

[54] Silva M.V.B., Alet A.I., Castellini H.V., Riquelme B.D. (2022). Methods: A new protocol for in vitro red blood cell glycation. Comp. Biochem. Physiol..

[55] Giustarini D., Fanti P., Matteucci E., Rossi R. (2014). Micro-method for the determination of glutathione in human blood. J. Chromatogr. B Anal. Technol. Biomed. Life Sci..

[56] Beutler E. (1975). Red Cell Metabolism: A Manual of Biochemical Methods.

[57] Franco R.R., da Silva Carvalho D., Rosa de Moura F.B., Justino A.B., Silva H.C.G., Peixoto L.G., Espindola F.S. (2018). Antioxidant and anti-glycation capacities of some medicinal plants and their potential inhibitory against digestive enzymes related to type 2 diabetes mellitus. J. Ethnopharmacol..

[58] Baker J.R., Zyzak D.V., Thorpe S.R., Baynes J.W. (1993). Mechanism of fructosamine assay: Evidence against role of superoxide as intermediate in nitroblue tetrazolium reduction. Clin. Chem..

[59] Levine R.I., Garland D., Oliver C.N., Amici A., Climent I., Lenz A.G., Ahn B.W., Shaltiel S., Stadtman E.R. (1990). Determination of carbonyl content in oxidatively modified proteins. Methods Enzymol..

[60] Ellman G.L. (1959). A colorimetric method for determining low concentrations of mercaptans. Arch. Biochem. Biophys..

[61] Justino A.B., Pereira M.N., Peixoto L.G., Vilela D.D., Caixeta D.C., de Souza A.V., Teixeira R.R., Silva H.C.G., Rosa de Moura F.B., Moraes I.B. (2017). Hepatoprotective properties of a polyphenol-enriched fraction from *Annona crassiflora* Mart. fruit peel against diabetes-induced oxidative and nitrosative stress. J. Agric. Food Chem..

[62] Wu X., Zhang G., Hu M., Pan J., Li A., Zhang Y. (2020). Molecular characteristics of gallocatechin gallate affecting protein glycation. Food Hydrocoll..

[63] Morris G.M., Huey R., Lindstrom W., Sanner M.F., Belew R.K., Goodsell D.S., Olson A.J. (2009). AutoDock4 and AutoDockTools4: Automated docking with selective receptor flexibility. J. Comput. Chem..

[64] Trott O., Olson A.J. (2010). AutoDock Vina: Improving the speed and accuracy of docking with a new scoring function, efficient optimization, and multithreading. J. Comput. Chem..

